# *Nme1* and *Nme2* genes exert metastasis-suppressor activities in a genetically engineered mouse model of UV-induced melanoma

**DOI:** 10.1038/s41416-020-01096-w

**Published:** 2020-10-07

**Authors:** Nidhi Pamidimukkala, Gemma S. Puts, M. Kathryn Leonard, Devin Snyder, Sandrine Dabernat, Edward C. De Fabo, Frances P. Noonan, Andrzej Slominski, Glenn Merlino, David M. Kaetzel

**Affiliations:** 1 Department of Biochemistry and Molecular Biology, School of Medicine, University of Maryland-Baltimore, Baltimore, MD USA; 2 INSERM U1035, Université de Bordeaux, Bordeaux, France; 3 The George Washington University Medical Center, Washington, DC USA; 4 Departments of Dermatology and Pathology, University of Alabama at Birmingham, Birmingham, AL USA; 5 Laboratory of Cancer Biology and Genetics, Center for Cancer Research, National Cancer Institute, Bethesda, MD USA; 6 Marlene and Stewart Greenebaum Comprehensive Cancer Center, University of Maryland-Baltimore, Baltimore, MD USA; 7 Research and Development Service, VA Maryland Health Care System, Baltimore, MD USA; 8 Present Address: American Association for Cancer Research, Philadelphia, PA USA

**Keywords:** Metastasis, Melanoma

## Abstract

*NME1* is a metastasis-suppressor gene (MSG), capable of suppressing metastatic activity in cell lines of melanoma, breast carcinoma and other cancer origins without affecting their growth in culture or as primary tumours. Herein, we selectively ablated the tandemly arranged *Nme1* and *Nme2* genes to assess their individual impacts on metastatic activity in a mouse model (HGF:p16^−/−^) of ultraviolet radiation (UVR)-induced melanoma. Metastatic activity was strongly enhanced in both genders of *Nme1-* and *Nme2*-null mice, with stronger activity in females across all genotypes. The study ascribes MSG activity to *Nme2* for the first time in an in vivo model of spontaneous cancer, as well as a novel metastasis-suppressor function to *Nme1* in the specific context of UVR-induced melanoma.

## Background

Metastasis-suppressor genes (MSGs) inhibit the metastatic activity of tumour cells in vitro and in vivo without impacting growth characteristics in culture or as xenografts in immune-deficient mice. *NME1* was the first MSG identified,^1^ with suppressor-like functions demonstrated in cultured cell lines of multiple cancer origins.^2^ Consistent with its putative metastasis-suppressor function, multiple studies have reported an association between low *NME1* expression and more aggressive forms of melanoma in human patients.^2^ In addition, we recently showed that human melanoma cell lines harbour a rare cell subpopulation that exhibits reduced *NME1* and *NME2* expression, and strong metastatic activity when xenografted in immunocompromised mice.^3^ NME1 and NME2 are nucleoside diphosphate kinases, whose activities appear to be effected autonomously and in conjunction with subcellular structures via protein–protein interactions.^4^ Disruption of mouse *Nme1* confers metastatic potential to chemical-induced hepatocellular carcinoma in vivo, providing evidence of MSG activity in a spontaneously generated cancer.^5^ To date, however, MSG activity has yet to be ascribed to *Nme1* in an in vivo model of spontaneously generated melanoma, or to *Nme2* in any such in vivo model of cancer. We previously showed that concomitant, hemizygous ablation of the tandemly arranged *Nme1* and *Nme2* genes (*Nme1*^*+/Δ*^: *Nme2*^*+/Δ*^) converts ultraviolet radiation (UVR)-induced melanomas to highly metastatic forms in mice engineered for overexpression of hepatocyte growth factor (HGF), but the individual contributions of *Nme1* and *Nme2* were not addressed.^6^ Herein, we assessed the individual MSG activities of *Nme1* and *Nme2* in UVR-induced melanoma by their selective inactivation in a refined HGF-based mouse model. This approach demonstrated robust MSG activity of *Nme2* for the first time in any model of spontaneous cancer, while revealing strong suppressor activity of *Nme1* in UVR-induced melanoma.

## Methods

### Mice, UV irradiation and assessments of tumour growth and metastasis

Transgenic mouse strains were in the C57BL/6 genetic background. The parental HGF, *Nme1/2*^+/Δ^, *Ink4a*/*p16*^−/−^ strains, including methodologies for genotype screening, have been described.^6–10^ Experimental protocols were approved by the Institutional Care and Use Committee at the University of Maryland-Baltimore (Protocols 0612013 and 0515008; D. Kaetzel, P.I.). Melanoma was initiated in male and female mice (postnatal day four) by exposure to an erythematous dose of UV radiation (9 kJ/M^2^), as described.^6^ *Assessment of subcutaneous melanomas*. Experimental mice were individually examined weekly for the initiation and growth of subcutaneous melanoma lesions over the course of the experiment. Dimensions of pigmented skin tumours were measured weekly, with tumour volume calculated using the formula π/6 x L x W.^2^ *Method of euthanasia.* Mice were killed using CO_2_ asphyxiation and cervical dislocation for the harvesting of primary and metastatic tissues. For CO_2_ asphyxiation, narcosis was induced by using the gradual fill method for CO_2_ gas administration by using a commercially available cylinder, pressure reducing regulator, and flow metre, or equivalent equipment. A euthanasia chamber that is equipped with a restriction valve (which is never removed) allowed the rate of CO_2_ gas flow to be within the 10–30% chamber volume per minute fill rate, as described in the 2013 AVMA Guidelines. CO_2_ flow was maintained for at least 1 min following respiratory arrest, which required 3–5 min. The cervical dislocation was performed secondarily to assure euthanasia. *Analysis of metastatic lesions.* Tissues from primary melanomas, left and right lymph nodes (cervical, axillary, brachial and inguinal), liver, and lung were dissected at necropsy. To calculate lung metastasis scores, pigmented lesions were counted and measured on dorsal surfaces of both lungs. Lesions were classified into four categories based on diameter and assigned different weights: <0.5 mm (1×), 0.5–1 mm (2×), 1–5 mm (3×) and >5 mm (4×). Metastasis scores represented the sum of lesion numbers × their weighting scores: *n* (1×) + *n* (2×) + *n* (3×) + *n* (4×). Lymph-node volumes were calculated as described above for primary melanomas and weighted (<10 mm^3^ = 1, 10–20 mm^3^ = 2, 20–30 mm^3^ = 3, etc.). Lymph-node enlargement scores represent sums of weighted scores for all nodes. The primary tumours and selected organs were processed for microscopic analysis, as we described previously.^6^

## Results

**Fig. 1:**
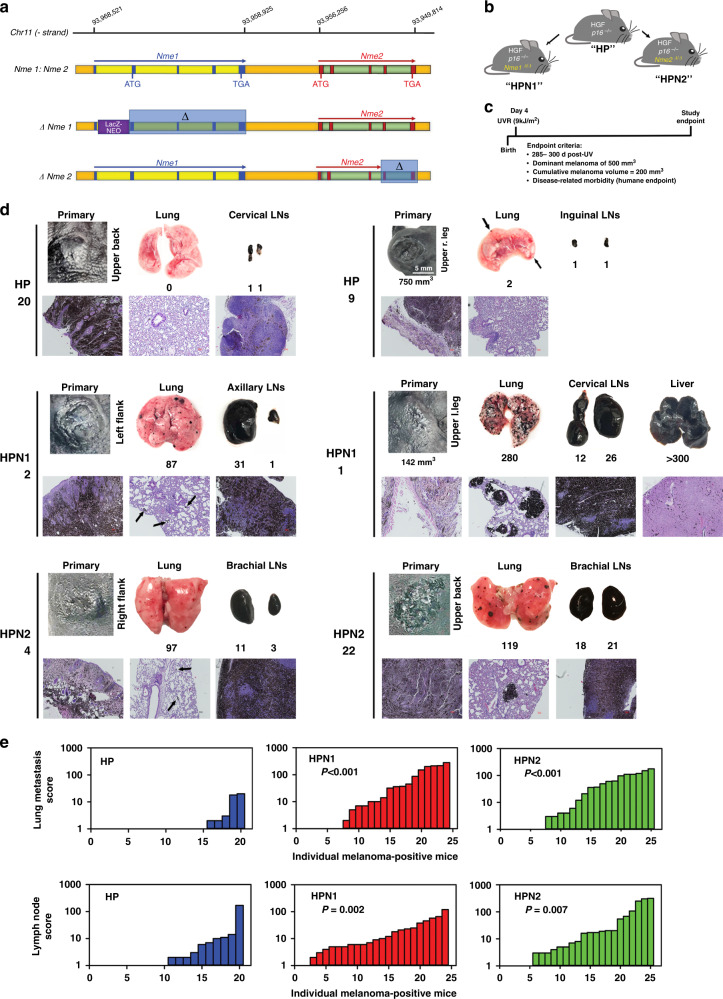
Genetic ablation of either *Nme1* or *Nme2* confers strong metastatic activity to UV-induced melanomas in hepatocyte growth factor (HGF): p16^−/−^ (HP) mice. **a** Organisation of the *Nme1* and *Nme2* genes and strategy for selective disruption of each in transgenic mouse strains. “*Nme1:Nme2*” depicts the genomic *Nme1*: *Nme2* locus in the mouse. The structural *Nme1* gene is denoted in yellow, with exons represented by blue squares. Translational initiation (ATG) and termination (TGA, TAG) codons are also identified within both genes. *Nme2* is denoted in green, with exons represented by brown squares. The sequence labelled “*ΔNme1*” portrays the insertion of a LacZ-NEO cassette within intron 1 of the *Nme1* gene, preventing transcription for downstream coding exons 2–4 (denoted in the diagram as Δ, and outlined by the translucent blue box) while sparing expression of *Nme2.*^8^ “*ΔNme2*” depicts the deletion of exons 4 and 5 within the *Nme2* gene, resulting in expression of a truncated transcript that is unstable and poorly translated.^9^ **b** Diagram illustrates the three strains of mice employed in the study. The “HP” strain was engineered for overexpression of HGF and homozygous deletion of the *Ink4a/p16* locus (p16^−/−^).^10^ Strains “HPN1” and “HPN2” represent hybrid crosses of the HP strain with mice harbouring either the Δ*Nme1* or Δ*Nme2* genotypes in the homozygous form (“*Nme1*^*Δ/Δ*^” or “*Nme2*^*Δ/Δ*^”), as shown. **c** Protocol for ultraviolet radiation (UVR) initiation and monitoring of melanomas in HP-based mouse strains. **d** Images of primary melanomas, lungs, and proximal lymph nodes from representative HP, HPN1 and HPN2 mice (numbers following HP/HPN genotype indicate mouse identifiers). Macroscopic photographs of tissues are displayed in the top set of three images for each mouse, with volumes of primary melanomas (1^o^), lung metastasis scores, and lymph-node enlargement scores provided below the corresponding images. Representative microscopic images of haematoxylin/eosin-stained tissue sections are shown below each corresponding macroscopic image (scale bars represent 50 μm). **e** Summaries of lung metastasis and lymph-node enlargement scores for all melanoma-positive HP, HPN1 and HPN2 mice. Lung metastasis and lymph-node scores for HPN1 and HPN2 mice were compared to those of HP mice using the Mann–Whitney rank-sum test, with probabilities displayed within the corresponding panels.

We previously demonstrated MSG activity of the *Nme1*/*Nme2* locus (Fig. 1a) using the HGF mouse model of UVR-induced melanoma.^6^ In this study, we assessed metastasis-suppressor activities of the individual *Nme1* and *Nme2* genes in UVR-induced melanoma using HGF mice harbouring an additional, homozygous-null deletion of the *Ink4a/p16* gene (**H**GF: ***P****16* ^−/−^ or “HP strain”). The p16^−/−^ lesion accelerates the onset of UVR-induced melanomas in HGF mice while retaining melanoma frequency and low metastatic activity.^11^ Mouse strains harbouring selective inactivation of either *Nme1* (Nme1^*Δ/Δ*^)^8^ or *Nme2* (*Nme2*^*Δ/Δ*^)^9^ (Fig. 1a) were crossed with HP mice, yielding the strains **H**GF: ***P****16* ^−/−^: ***N****me****1***^*Δ/Δ*^ (“HPN1”) and **H**GF: ***P****16* ^−/−^: ***N****me****2***^*Δ/Δ*^ (“HPN2”), respectively (Fig. 1b).

Melanoma was initiated in male and female HP, HPN1 and HPN2 mice with UVR (9 kJ/M^2^) to the dorsal skin at postnatal day 4. Incidence and growth of melanomas were monitored bi-weekly until the attainment of a study endpoint (Fig. 1c). Melanoma incidence was high in all three strains (>90%), with most mice exhibiting a low frequency of tumours/mouse (1–4 lesions; Supplementary Table 1). All melanomas displayed strong melanin pigmentation, with most gradually acquiring a domed appearance over the course of their growth and central necrosis/ulceration as they approached critical size (500 mm^3^; Fig. 1d). Most melanomas were located on exposed dorsal skin surfaces (back, neck, top of head and flanks), although some were detected on the extremities (legs, tail and ears) and face (Supplementary Table 1). Histopathological analysis of representative UVR-induced primary and metastatic melanomas of all three strains revealed (Fig. 1d) characteristics identical to those previously described for HGF mice with wild-type or hemizygous-null genotypes at the *Nme1:Nme2* locus.^6^ All melanoma specimens harboured varying ratios of both epithelioid and dendritic melanoma cells that were highly pigmented (Supplementary Table 2).

HPN2 mice exhibited more than double the incidence of face tumours (8.3%) than HP (2.9%) or HPN1 (3.4%) mice. Melanoma onset was similar for HP (median: 136 d post-UV), HPN1 (147 d) and HPN2 (113 d) mice (Supplementary Table 3), but was markedly earlier than seen previously in HGF mice (~200 d).^6^ The three strains exhibited small differences in the number of primary melanomas/mouse (HP > HPN1 = HPN2), rates of growth for the largest primary melanoma (HPN2 > HP) and final combined tumour volume (HPN2 > HP). No other differences were attributable to *Nme* genotype or gender, however, in growth rates for individual melanomas, days between onset and study endpoint, size of the largest melanoma/mouse, or final combined tumour volume/mouse (Supplementary Table 3).

Melanoma-positive HPN1 and HPN2 mice displayed much higher incidence and overall scores for lung metastasis and lymph-node enlargement than HP mice (Fig. 1d, e and Supplementary Table 3). Lung metastasis and lymph-node enlargement scores were highly correlated across all genotypes (*P* < 2e-7), providing validation of the respective scoring systems. None of the characteristics of primary melanoma growth monitored in the study were correlated with lung metastasis or lymph-node enlargement (Supplementary Table 3). Lung metastasis was enhanced in both male and female HPN1 and HPN2 mice, with females exhibiting higher lung metastasis and lymph-node enlargement scores across all genotypes (Supplementary Table 3). Effects of gender and genotype on metastasis scores were independent.

## Discussion

This study demonstrates that the ablation of either *Nme1* or *Nme2* confers robust increases in metastatic activity in the HGF-based mouse model of UV-induced melanoma. Increased metastatic activity in HPN1 and HPN2 mice was associated with *Nme1* or *Nme2* inactivation and was not secondary to growth characteristics of melanomas or tumour burden (e.g., size or number). Together, these findings provide the first demonstration of metastasis-suppressor activity for *Nme1* in an in vivo context of spontaneous melanoma. Moreover, they establish the metastasis-suppressor function of *Nme2* for the first time in an in vivo model of spontaneous cancer of any kind, thereby suggesting a similar function for the human *NME2* gene. To date, assessments of human *NME2* have used only cultured cell lines of various cancer origins and measurements of surrogate phenotypes of metastasis, such as motility and invasiveness in cell culture systems or metastatic growth of cell xenografts in immunocompromised mice. ^12,13^ The robust metastatic activity observed in HPN1 and HPN2 mice would appear to render the strains ideal for the discovery of novel prognostic markers and therapeutic targets in the management of advanced melanoma. In particular, NextGen sequencing of primary and metastatic melanoma lesions in HPN1 and HPN2 mice could be leveraged to identify genomic and transcriptomic signatures associated with the metastatic activity, and by extension, potential correlations with metastatic activity and survival in human melanoma patients.

Predominant melanomas of HPN2 mice grew at a modestly higher rate than those of HP or HPN1 mice, suggesting *Nme2* has a non-canonical suppressive effect on the proliferation of melanoma cells in vivo. Nevertheless, growth rates of primary tumours in HPN2 mice were not correlated with lung metastasis or lymph-node enlargement scores, indicating the impact of *Nme2* ablation on metastasis was independent of its effect on primary tumour growth. The higher rate of face tumours in HPN2 versus HP or HPN1 mice suggests *Nme2* deficiency imparts a more external and UV-exposed location of melanocytes in face skin, or that *Nme2* might play a more dominant role than *Nme1* in the repair of UV-induced DNA damage in those melanocytes. Our findings obtained after selective ablation of *Nme1* and *Nme2* do not exclude the possibility that *Nme1* and *Nme2* may still act in concert to suppress metastasis. In addition, we have observed coordinate downregulation of *NME1* and *NME2* in human melanoma cell lines of metastatic origin,^14^ and this has been reported in human cervical carcinoma specimens as well,^15^ suggesting cooperative impacts of *NME1* and *NME2* on metastatic activity. Moreover, a recent study demonstrated that NME1 and NME2 proteins are found almost exclusively in the form of mixed oligomers, suggesting that they may possess complementary functions.^16^ Expression of a human *NME1–NME2* fusion gene has been reported^17^ but is expressed at much lower levels than the individual *NME1* and *NME2* genes, suggesting its impact on metastasis is minor. We recently showed that human melanoma cell lines harbour a rare subpopulation of cells that are profoundly deficient in expression of NME1 and NME2 and exhibit greatly enhanced metastatic activity.^3^ By virtue of their complete ablation of *Nme1* and *Nme2* expression, HPN1 and HPN2 mice are relevant in vivo models for the NME-deficient and highly metastatic subpopulation in human cells.

Overall, the robust metastasis-suppressor activities exhibited in the current study by both *Nme1* and *Nme2* provide novel and compelling in vivo evidence for the individual roles of these genes in malignant progression. In turn, the findings obtained in our mouse models offer a critical rationale for continued efforts to identify metastasis-driving pathways under the suppressive influence of *NME1* and *NME2* in humans.

## Supplementary information

Supplementary Table 1

Supplementary Table 2

Supplementary Table 3

## Data Availability

Data supporting the findings of this study are available within the article and its supplementary files. Other relevant data and information such as breeding schemes, photographic images of primary and metastatic melanoma tissues, and assessments of melanoma growth in individual mice are available from the corresponding author upon reasonable request.
