# Supplementary material for: Nme1 and Nme2 genes exert metastasis-suppressor activities in a genetically engineered mouse model of UV-induced melanoma
Source: Br J Cancer. 2020 Oct 7;124(1):161–5. doi: 10.1038/s41416-020-01096-w (PMC7782836; doi:10.1038/s41416-020-01096-w)
Supplement: Supplementary file 1 — Supplementary Table 1 [file 41416_2020_1096_MOESM1_ESM.pdf]

Supplementary Table 1. Statistical comparisons of gender distribution, tumor location and metastasis incidence

A. Gender distribution in HP, HPN1 and HPN2 mice

						Chi-square P-Value	
**Genotype**	**Total Mice**	**Male**	**Female**	**% Male**	**% Female**	**HP vs HPN1**	**HP vs HPN2**
HP	20	14	6	70.0	30.0		
HPN1	24	15	9	62.5	37.5	0.102	
HPN2	25	8	17	32.0	68.0		< 0.001

B. Anatomical locations of primary melanomas in HP, HPN1 and HPN2 mice

	Back/Neck/Top of head/Flanks		Extremities: Hindlegs/ Forelegs/Tail/Ears		Face: Cheeks/ Nose		Chi-square P-Value	
**Genotype**	**Total**	**%**	**Total**	**%**	**Total**	**%**	**HP vs HPN1**	**HP vs HPN2**
HP	59	85.5	8	11.6	2	2.9		
HPN1	48	82.8	8	13.8	2	3.4	0.359	
HPN2	48	80.0	7	11.7	5	8.3		0.036

C. Incidence of primary face melanomas in HP, HPN1 and HPN2 mice

			Chi-square P-Value	
**Genotype**	**Face tumor positive**	**Face tumor negative**	**HP vs HPN1**	**HP vs HPN2**
HP	2	18		
HPN1	2	22	0.346	
HPN2	5	20		0.030

D. Incidence of lung metastases in HP, HPN1 and HPN2 mice

**Genotype**	**Total Mice**	**Metastasis-positive**	**Metastasis-negative**	**% Metastasis-positive**	**% Metastasis-negative**	Chi-square P-Value	
						**HP vs HPN1**	**HP vs HPN2**
HP	20	5	15	25.0	75.0		
HPN1	24	18	6	75.0	25.0	< 0.001	
HPN2	25	18	7	72.0	28.0		< 0.001

E. Incidence of lymph node enlargement in HP, HPN1 and HPN2 mice

**Genotype**	**Total Mice**	**Enlargement-positive**	**Enlargement-negative**	**% Enlargement-positive**	**% Enlargement-negative**	Chi-square P-Value	
						**HP vs HPN1**	**HP vs HPN2**
HP	20	13	15	25.0	75.0		
HPN1	24	22	2	91.7	8.3	< 0.001	
HPN2	25	21	4	84.0	16.0		< 0.001
