# Supplementary material for: Nme1 and Nme2 genes exert metastasis-suppressor activities in a genetically engineered mouse model of UV-induced melanoma
Source: Br J Cancer. 2020 Oct 7;124(1):161–5. doi: 10.1038/s41416-020-01096-w (PMC7782836; doi:10.1038/s41416-020-01096-w)
Supplement: Supplementary file 2 — Supplementary Table 2 [file 41416_2020_1096_MOESM2_ESM.pdf]

Supplementary Table 2. Histological characteristics of primary melanomas and metastases

Mouse ID	Tissue	Histological Characteristics
HP9	Primary melanoma	Epithelioid and dendritic heavily pigmented melanoma cells
	Right axillary lymph node	Clusters of epithelioid heavily pigmented melanoma cells
HP20	Primary melanoma	Epithelioid and dendritic heavily pigmented melanoma cells
	Left cervical lymph node	Epithelioid and dendritic heavily pigmented melanoma cells
	Lung	Negative
HPN1-1	Primary melanoma	Mainly dendritic with some epithelioid heavily pigmented melanoma cells
	Left cervical lymph node	Epithelioid and dendritic heavily pigmented melanoma cells
	Lung	Clusters of epithelioid heavily pigmented melanoma cells
	Liver	Clusters of epithelioid heavily pigmented melanoma cells
HPN1-2	Primary melanoma	Epithelioid and dendritic heavily pigmented melanoma cells
	Left axillary lymph node	Replaced by epithelioid and dendritic heavily pigmented cells
	Lung	Microscopic foci of epithelioid heavily pigmented cells
HPN2-4	Primary melanoma	Mainly dendritic with some epithelioid heavily pigmented melanoma cells
	Left cervical lymph node	Replaced by epithelioid and dendritic heavily pigmented cells
	Lung	Microscopic foci of epithelioid heavily pigmented cells
HPN2-22	Primary melanoma	Mainly dendritic with some epithelioid heavily pigmented melanoma cells
	Left brachial lymph node	Replaced by epithelioid and dendritic heavily pigmented cells
	Lung	Microscopic foci of epithelioid heavily pigmented cells
