# Supplementary material for: Nme1 and Nme2 genes exert metastasis-suppressor activities in a genetically engineered mouse model of UV-induced melanoma
Source: Br J Cancer. 2020 Oct 7;124(1):161–5. doi: 10.1038/s41416-020-01096-w (PMC7782836; doi:10.1038/s41416-020-01096-w)
Supplement: Supplementary file 3 — Supplementary Table 3 [file 41416_2020_1096_MOESM3_ESM.pdf]

Supplementary Table 3. Statistical comparisons of tumor growth characteristics and metastasis scores

A. Comparisons of tumor growth characteristics and metastasis scores for HP vs HPN1 mice

Variable	Test*	HP n	HPN1 n	HP Mean	HP SEM	HPN1 Mean	HPN1 SEM	P-Value	HP Median	HP 25%	HP 75%	HPN1 Median	HPN1 25%	HPN1 75%	P-Value
Tumor onset days post-irradiation	*t* -test	20	24	135.5	9.9	147.2	11.6	0.460							
Days between onset and endpoint	*t* -test	20	24	121.3	11.7	100.7	9.4	0.173							
Number of primary tumors monitored for growth	Rank sum test	20	24						3.0	1.0	4.0	2.0	1.0	2.0	0.036
Largest tumor at necropsy volume mm^3	Rank sum test	20	24						70.0	48.2	191.6	258.3	70.4	638.9	0.061
Slope of growth curve for largest tumor: (tumor volume)/Days post-UV	Rank sum test	20	24						0.0083	0.0058	0.0154	0.0125	0.0077	0.0166	0.263
Final Combined Tumor Volume mm^3	Rank sum test	20	24						210.4	125.5	402.9	318.6	115.9	770.9	0.225
Lung metastasis score	Rank sum test	20	24						0.0	0.0	1.5	10.0	0.3	76.5	< 0.001
Composite lymph node volume score	Rank sum test	20	24						1.5	0.0	6.8	8.0	5.0	24.5	0.002

B. Comparisons of tumor growth characteristics and metastasis scores for HP vs HPN2 mice

Variable	Test*	HP n	HPN2 n	HP Mean	HP SEM	HPN2 Mean	HPN2 SEM	P-Value	HP Median	HP 25%	HP 75%	HPN2 Median	HPN2 25%	HPN2 75%	P-Value
Tumor onset days post-irradiation	*t* -test	20	25	135.5	9.9	112.6	10.5	0.126							
Days between onset and endpoint	*t* -test	20	25	121.3	11.7	86.8	10.0	0.030							
Number of primary tumors monitored for growth	Rank sum test	20	25						3.0	1.0	4.0	1.0	1.0	2.0	0.033
Largest tumor at necropsy volume mm^3	Rank sum test	20	25						70.0	48.2	191.6	357.1	63.8	478.9	0.073
Slope of growth curve for largest tumor: (tumor volume)/Days post-UV	Rank sum test	20	25						0.0083	0.0058	0.0154	0.0154	0.0113	0.0361	0.014
Final Combined Tumor Volume mm^3	Rank sum test	20	25						210.4	125.5	402.9	559.4	249.4	1009.8	0.028
Lung metastasis score	Rank sum test	20	25						0.0	0.0	1.5	12.0	0.0	79.5	< 0.001
Composite lymph node volume score	Rank sum test	20	25						1.5	0.0	6.8	8.0	3.0	37.0	0.007

C. Comparisons of tumor growth characteristics and metastasis scores for male vs female mice across all genotypes

Variable	Test*	Male n	Female n	Male Mean	Male SEM	Female Mean	Female SEM	P-Value	Male Median	Male 25%	Male 75%	Female Median	Female 25%	Female 75%	P-Value
Tumor onset days post-irradiation	Rank sum test	37	32						148	92.00	173.0	104.50	78.750	168.5	0.188
Days between onset and endpoint	*t* -test	37	32	114.6	8.9	86.7	7.5	0.021							
Number of primary tumors monitored for growth	Rank sum test	37	32						2.0	1.0	3.5	1.0	1.0	2.0	0.006
Largest tumor at necropsy volume mm^3	Rank sum test	37	32						198.5	82.6	425.4	153.9	40.2	613.3	0.661
Slope of growth curve for largest tumor: (tumor volume)/Days post-UV**	Rank sum test	37	32						0.00929	0.00654	0.0156	0.0148	0.0113	0.0295	0.005
Final Combined Tumor Volume mm^3	Rank sum test	37	32						343.7	210.1	800.8	259.0	65.4	670.3	0.134
Lung metastasis score	Rank sum test	37	32						0.0	0.0	10.0	20.5	2.3	80.8	< 0.001
Composite lymph node volume score	Rank sum test	37	32						4.0	1.0	9.5	14.0	2.3	57.0	0.008

D. Comparisons of tumor growth characteristics and metastasis scores for male HP vs HPN1 mice

Variable	Test*	HP n	HPN1 n	HP Mean	HP SEM	HPN1 Mean	HPN1 SEM	P-Value	HP Median	HP 25%	HP 75%	HPN1 Median	HPN1 25%	HPN1 75%	P-Value
Tumor onset days post-irradiation	*t* -test	14	15	133.9	10.9	154.6	14.5	0.271							
Days between onset and endpoint	*t* -test	14	15	136.1	14.7	93.9	12.2	0.035							
Number of primary tumors monitored for growth	Rank sum test	14	15						3.5	1.75	4.0	2.0	1.0	3.0	0.043
Largest tumor at necropsy volume mm^3	Rank sum test	14	15						82.6	46.9	161.1	357.3	150.0	669.0	0.012
Slope of growth curve for largest tumor: (tumor volume)/Days post-UV	Rank sum test	14	15						0.0079	0.0055	0.0149	0.0124	0.0064	0.0167	0.419
Final Combined Tumor Volume mm^3	Rank sum test	14	15						210.4	164.1	354.3	390.3	258.4	849.2	0.052
Lung metastasis score	Rank sum test	14	15						0.0	0.0	0.0	5.0	0.0	32.0	0.005
Composite lymph node volume score	Rank sum test	14	15						1.0	0.0	4.0	6.0	4.0	18.0	0.013

E. Comparisons of tumor growth characteristics and metastasis scores for male HP vs HPN2 mice

Variable	Test*	HP n	HPN2 n	HP Mean	HP SEM	HPN2 Mean	HPN2 SEM	P-Value	HP Median	HP 25%	HP 75%	HPN2 Median	HPN2 25%	HPN2 75%	P-Value
Tumor onset days post-irradiation	*t* -test	14	8	133.9	10.9	121.0	19.3	0.535							
Days between onset and endpoint	*t* -test	14	8	136.1	14.7	115.6	20.3	0.417							
Number of primary tumors monitored for growth	*t* -test	14	8	3.0	0.3	1.8	0.4	0.031							
Largest tumor at necropsy volume mm^3	Rank sum test	14	8						82.6	46.9	161.1	371.3	156.5	444.0	0.005
Slope of growth curve for largest tumor: (tumor volume)/Days post-UV	Rank sum test	14	8						0.0079	0.0055	0.0149	0.0111	0.0073	0.0147	0.357
Final Combined Tumor Volume mm^3	Rank sum test	14	8						210.4	164.1	354.3	798.8	444.1	1152.4	0.004
Lung metastasis score	Rank sum test	14	8						0.0	0.0	0.0	1.5	0.0	73.8	0.029
Composite lymph node volume score	Rank sum test	14	8						1.0	0.0	4.0	4.5	3.0	14.5	0.049

F. Comparisons of tumor growth characteristics and metastasis scores for face tumor-positive vs face tumor-negative mice across all genotypes

Variable	Test*	Face tumor-positive n	Face tumor-negative n	Face tumor-positive Mean	Face tumor-positive SEM	Face tumor-negative Mean	Face tumor-negative SEM	P-Value	Face tumor-positive Median	Face tumor-positive 25%	Face tumor-positive 75%	Face tumor-negative Median	Face tumor-negative 25%	Face tumor-negative 75%	P-Value
Tumor onset days post-irradiation	Rank sum test	9	60						129.5	82.0	168.5	127.0	95.0	198.5	0.769
Days between onset and endpoint	*t* -test	9	60	63.0	10.7	107.4	6.5	0.013							
Number of primary tumors monitored for growth	Rank sum test	9	60						1.0	1.0	3.0	2.0	1.0	3.0	0.635
Largest tumor at necropsy volume mm^3	Rank sum test	9	60						145.8	63.2	388.1	191.9	50.4	553.3	0.515
Slope of growth curve for largest tumor: (tumor volume)/Days post-UV	Rank sum test	9	60						0.013	0.010	0.028	0.012	0.007	0.018	0.418
Final Combined Tumor Volume mm^3	Rank sum test	9	60						261.0	68.7	408.7	312.0	169.5	807.6	0.236
Lung metastasis score	Rank sum test	9	60						110.0	1.0	188.0	3.0	0.0	29.3	0.028
Composite lymph node volume score	Rank sum test	9	60						107.0	22.5	208.5	5.0	1.0	15.5	0.001

G. Comparisons of tumor growth characteristics and metastasis scores for face tumor-negative HP vs HPN1 mice

Variable	Test*	HP n	HPN1 n	HP Mean	HP SEM	HPN1 Mean	HPN1 SEM	P-Value	HP Median	HP 25%	HP 75%	HPN1 Median	HPN1 25%	HPN1 75%	P-Value
Tumor onset days post-irradiation	*t* -test	18	22	132.7	10.5	142.1	12.1	0.570							
Days between onset and endpoint	*t* -test	18	22	126.6	12.4	105.1	9.7	0.173							
Number of primary tumors monitored for growth	Rank sum test	18	22						3.0	1.0	4.0	2.0	1.0	2.3	0.042
Largest tumor at necropsy volume mm^3	Rank sum test	18	22						67.8	46.9	209.1	258.3	83.2	661.6	0.084
Slope of growth curve for largest tumor: (tumor volume)/Days post-UV	Rank sum test	18	22						0.0079	0.0055	0.0149	0.0118	0.0073	0.0164	0.271
Final Combined Tumor Volume mm^3	Rank sum test	18	22						210.4	145.5	409.6	318.6	162.0	802.8	0.216
Lung metastasis score	Rank sum test	18	22						0.0	0.0	0.5	8.5	0.0	39.0	0.001
Composite lymph node volume score	Rank sum test	18	22						1.0	0.0	3.8	6.5	4.8	21.5	< 0.001

H. Comparisons of tumor growth characteristics and metastasis scores for face tumor-negative HP vs HPN2 mice

Variable	Test*	HP n	HPN2 n	HP Mean	HP SEM	HPN2 Mean	HPN2 SEM	P-Value	HP Median	HP 25%	HP 75%	HPN2 Median	HPN2 25%	HPN2 75%	P-Value
Tumor onset days post-irradiation	Rank sum test	18	20						143.0	91.3	163.3	86.0	69.8	175.3	0.365
Days between onset and endpoint	*t* -test	18	20	126.6	12.4	92.7	11.5	0.052							
Number of primary tumors monitored for growth	Rank sum test	18	20						3.0	1.0	4.0	1.0	1.0	2.0	0.038
Largest tumor at necropsy volume mm^3	Rank sum test	18	20						67.8	46.9	209.1	371.3	82.6	543.3	0.093
Slope of growth curve for largest tumor: (tumor volume)/Days post-UV	Rank sum test	18	20						0.0079	0.0055	0.0149	0.0156	0.0114	0.0430	0.007
Final Combined Tumor Volume mm^3	Rank sum test	18	20						210.4	145.5	409.6	764.2	254.8	1119.1	0.023
Lung metastasis score	Rank sum test	18	20						0.0	0.0	0.5	5.0	0.0	57.3	0.001
Composite lymph node volume score	Rank sum test	18	20						1.0	0.0	3.8	6.0	3.0	18.5	0.005

I. Correlations of tumor growth characteristics and metastasis scores across all HP, HPN1 and HPN2 mice**

Variable	n	Lung metastasis score		Lymph node enlargement score	
		r	P-Value	*r*	P-Value
Tumor onset days post-irradiation	69	-0.003	0.982	0.080	0.512
Days between onset and endpoint	69	-0.102	0.401	0.011	0.931
Number of primary tumors monitored for growth	69	-0.080	0.515	-0.034	0.780
Largest tumor at necropsy volume mm^3	69	0.140	0.251	-0.072	0.553
Slope of growth curve for largest tumor: (tumor volume)/Days post-UV	69	0.146	0.232	0.001	0.995
Final Combined Tumor Volume mm^3	69	0.123	0.314	-0.059	0.628
Lung metastasis score	69			0.740	< 0.001

* The parametric Student's *t* -test was used to compare variables that were normally distributed, and the non-parametric Mann-Whitney rank sum test was used to test variables that were not normally distributed.

** The non-parametric Spearman correlation test was used for all analyses.
